# Three-year prevalence of healthcare-associated infections in Dutch nursing homes

**DOI:** 10.1186/1753-6561-5-S6-P158

**Published:** 2011-06-29

**Authors:** A Eikelenboom-Boskamp, J Cox-Claessens, P Boom-Poels, M Drabbe, R Koopmans, A Voss

**Affiliations:** 1 Medical Microbiology , Radboud University Medical Centre, Netherlands; 2 Medical Microbiology and Infectious Diseases, Canisius Wilhelmina Hospital, Netherlands; 3 ZZG Zorggroep, nursing home Margriet, Nijmegen, Netherlands; 4 Zorgcentra Pantein, nursing home Madeleine, Boxmeer, Netherlands; 5 Zorggroep Maas en Waal, nursing home Waelwick, Ewijk, Netherlands; 6 Primary and Community Care, Radboud University Medical Centre, Nijmegen, Netherlands

## Introduction / objectives

The objective of this study was to measure the prevalence of HAIs (overall as well as per type of care) and to gain insight into infection prevention and control and antimicrobial use in Dutch nursing homes.

## Methods

We conducted an annual, one-day prevalence study of HAIs among nursing home residents in the Nijmegen region over three consecutive years (2007-2009). Modified definitions based on CDC-criteria were used for bloodstream infection, lower respiratory tract infection, bacterial conjunctivitis and gastroenteritis. For urinary tract infections (UTI), criteria established by the Dutch Association of Elderly Care Physicians were used. Also resident characteristics were recorded. Data collection and resident assessment were done by the attending elderly care physicians.

## Results

The prevalence of HAIs over the three years was 6.7%, 7.6% and 7.6%, respectively. UTI was the most prevalent HAI with an overall prevalence of 3.8% and most UTI were not catheter-asscociated. Overall most HAIs occurred among residents of rehabilitation units. On average, antibiotics were used in 6.6% of the residents.

## Conclusion

This is the first prevalence study of HAIs in Dutch nursing homes, which became an important cornerstone of infection control programs in nursing homes, started in our region (see additional abstract). Based on the prevalence results, incidence studies for UTI need to be done in order to find risk-factors for their occurrence.

## Disclosure of interest

None declared.

